# A protocol for investigation of the effects of outdoor air pollution on stroke incidence, phenotypes and survival using the South London Stroke Register

**DOI:** 10.1186/1476-072X-5-10

**Published:** 2006-03-17

**Authors:** Ravi Maheswaran, Tim Pearson, Michael J Campbell, Robert P Haining, Cameron W McLeod, Nigel Smeeton, Charles DA Wolfe

**Affiliations:** 1 Public Health GIS Unit, School of Health and Related Research, University of Sheffield, Regent Court, 30 Regent Street, Sheffield S1 4DA, UK; 2 Health Services Research Unit, School of Health and Related Research, University of Sheffield, Regent Court, 30 Regent Street, Sheffield S1 4DA, UK; 3 Department of Geography, University of Cambridge, Downing Place, Cambridge CB2 3EN, UK; 4 Centre for Analytical Sciences, Department of Chemistry, University of Sheffield, Western Bank, Sheffield S10 2TN, UK; 5 Department of Public Health Sciences, Division of Health and Social Care Research, King's College, London, 5th Floor Capital House, 42 Weston Street, London SE1 3QD, UK

## Abstract

Stroke is a major cause of death and disability. About 5.3 million people die every year from stroke worldwide with over 9 million people surviving at any one time after suffering a stroke. About 1 in 4 men and 1 in 5 women aged 45 years will suffer a stroke if they live to their 85th year. It is estimated that by 2023 there will be an absolute increase in the number of people experiencing a first ever stroke of about 30% compared with 1983.

In the UK, stroke is the third commonest cause of death and the most common cause of adult physical disability and consumes 5% of the health and social services budget. Stroke is assuming strategic public health importance because of increased awareness in society, an ageing population and emerging new treatments. It is an NHS health service and research priority, being identified as a target in *Our Healthier Nation *and the *NSF for Older People *for prevention and risk factor control and in the *NHS Plan *as a disease requiring intermediate care planning and reduction in inequalities of care.

Whilst a number of risk factors for stroke are well known (e.g. increasing age, ethnicity, socioeconomic deprivation, hypertension), the potential importance of outdoor air pollution as a modifiable risk factor is much less well recognised. This is because studies to date are inconclusive or have methodological limitations. In Sheffield, we estimated that 11% of stroke deaths may be linked to current levels of outdoor air pollution and this high figure is explained by the fact that so many people are exposed to air pollution.

We plan to study the effects of outdoor air pollution on stroke using a series of epidemiological (i.e. population based) studies. The purpose of this project is:

• to examine if short term increases in pollution can trigger a stroke in susceptible individuals;

• to investigate if the occurrence of stroke is higher amongst people living in more polluted areas (which would be explained by a combination of exposure to short term increases and longer term exposure to higher pollution levels); and

• to see if people living in more polluted areas have reduced survival following their stroke.

We will use geographical information systems, robust statistical methods and powerful grid computing facilities to link and analyse the data. The datasets we will use are the South London Stroke Register database, daily monitored pollution data from national monitoring networks and modelled pollution data for London from the Greater London Authority. The South London Stroke Register records information on all patients who suffer a stroke ("incident" cases) living within a defined area. This stroke incidence dataset offers major advantages over previous studies examining the effects of pollution on hospital admissions and mortality, as not all patients with stroke are admitted or die and there may be a delay between the onset of stroke and admission or death. In addition, it contains other useful information, particularly the type of stroke people have suffered.

Air pollution is a potentially modifiable risk factor for stroke. This study will provide robust population level evidence regarding the effects of outdoor air pollution on stroke. If it confirms the link, it will suggest to policy-makers at national and international levels that targeting policy interventions at high pollution areas may be a feasible option for stroke prevention.

## Purpose of proposed investigation

There is increasing interest in the association between outdoor pollution and stroke and growing recognition that it may be a more important factor in terms of population attributable risk than previously recognised. To date, however, studies have been relatively small scale in size with inconclusive results and have not examined in detail the differential effects on stroke phenotypes. The purpose of this project is to examine the acute effects of outdoor air pollution on incident stroke and stroke phenotypes, investigate stroke incidence rates in high pollution areas which would be due to a combination of acute and chronic exposure effects, and examine if high outdoor air pollution levels reduce survival following stroke.

We plan to use case-crossover methodology, Bayesian hierarchical methods for spatial studies and survival analysis to examine these issues. The datasets we will use are the South London Stroke Register data which records incident stroke cases, daily monitored pollution data from national monitoring networks and modelled pollution data for London.

It is estimated that there are 5.3 million deaths a year from stroke worldwide and over 9 million survivors at any one time [1]. Almost one in four men and one in five women aged 45 will have a stroke if they live to their 85th year. The overall incidence is around 2 to 5 per thousand population. It is estimated that by 2023 there will be an absolute increase in the number of patients experiencing a first ever stroke of about 30% compared with 1983. There is a total prevalence rate of around 5 per thousand [1].

Stroke is the third most common cause of death and most common cause of adult physical disability in the UK and consumes 5% of the health and social services budget. Stroke is assuming strategic public health importance because of increased awareness in society, an ageing population and emerging new treatments. It is an NHS health service and research priority, being identified as a target in *Our Healthier Nation *and the *NSF for Older People *for prevention and risk factor control and in the *NHS Plan *as a disease requiring intermediate care planning and reduction in inequalities of care.

Whilst a number of risk factors for stroke are well known (e.g. age, ethnicity, deprivation, hypertension), the potential importance of outdoor air pollution as a modifiable risk factor is much less well recognised. We calculated that in Sheffield, 11% of stroke deaths may be attributable to outdoor air pollution and this high figure is explained by the fact that outdoor air pollution is a prevalent exposure [2]. Many of the numerous studies on the acute health effects of outdoor air pollution have not specifically examined stroke. In contrast, two high profile case-crossover studies [3,4] examining 772 and 691 myocardial infarction cases respectively, have contributed to establishing outdoor air pollution as a recognised trigger for myocardial infarction.

## Background to the programme of work

### Acute effects of air pollution on stroke

Early studies examining severe pollution episodes reported increased mortality from stroke [5,6], although one report found no increase in requests for hospital admission for cerebral haemorrhage [7]. Whilst pollution levels have reduced substantially, numerous daily time series studies have found associations between current levels of outdoor air pollutants and total and cardiorespiratory mortality. A number of studies on daily air pollution have specifically reported on stroke mortality, with several finding an association [8-13] but not others [14,15]. Similarly, several studies examining daily hospital admissions for stroke have observed adverse effects [16-20] while others have not [21,22]. However, reporting bias, i.e. an increased likelihood of reporting positive studies, cannot be ruled out. Several studies used time series analyses, while one used a case-crossover study design [20]. The pollutants for which studies have found associations include sulphur dioxide (SO_2_), nitrogen oxides (NO_x_), carbon monoxide (CO), ozone (O_3_) and particulate matter (PM_10_). However, studies reporting no evidence of association have also examined these pollutants. In two pollutant models examining stroke admissions using a case-crossover approach, Tsai [20] reported that the effects were mainly seen for PM_10 _and NO_2_, while Kan [11] encountered collinearity problems in multiple pollutant models in a time series study.

One study specifically examined sex differences in the air pollution effect and found that its effect on stroke mortality was only seen in women [10]. With regard to age, a study reported that the association with stroke mortality was only seen in people aged over 65 years [10] while another found associations with stroke admissions in people aged 20–64 and 65+ years [18]. The effect of NO_2 _and PM_10 _on stroke admissions was only apparent in warm weather (>20°C) in one study [20] while in another, an NO_2 _effect was observed in warm and cool weather while a CO effect was only seen in warm weather [19]. There is a strong association between socioeconomic deprivation and stroke [23] but effect modification by deprivation on the association between air pollution and stroke has not been examined. With respect to potential mechanisms, one hypothesis is that air pollution causes increased coagulability [24], which could explain ischaemic stroke. One study reported a significant association with only ischaemic stroke mortality [12] whilst another reported associations with both haemorrhagic and ischaemic stroke admissions [20]. However, no study has examined the association with clinical or aetiological phenotypes. We propose to examine the above issues using the South London Stroke Register dataset. This stroke incidence dataset offers major advantages over studies examining acute effects of pollution on hospital admissions and mortality, as not all patients with stroke are admitted or die and there may be a delay between stroke onset and admission or death.

### Stroke incidence rates in high pollution areas

Few studies have examined stroke mortality and hospital admission rates, and none have studied incidence rates, in areas with high levels of outdoor air pollution. In a national study, we found that stroke mortality risk was 5% higher in areas within 200 m of a main road, the latter used as a proxy for road traffic pollution [25]. We estimated that 990 stroke deaths per year in England and Wales would have been attributable to road traffic pollution if a causal link were assumed. In a small area level ecological correlation study we recently completed in Sheffield, we found that stroke mortality in the most polluted fifth of the city was 37% higher than that in the least polluted fifth of the city [2]. Overall, 11% of stroke deaths would have been attributable to outdoor air pollution. Elevated stroke mortality rates associated with outdoor air pollution in these studies would be due to a combination of its acute and chronic exposure effects.

We also found an association with stroke emergency hospital admission rates but the association was not as strong (13% increase in rate ratio for NO_x_) [2]. This may be because the spatial variation, even for emergency admissions, is influenced by a variety of factors including local medical practice and bed availability, and these could have masked the magnitude of the underlying association. Investigation of the association between outdoor air pollution and stroke incidence rates, as we propose to do in this project, should not be affected by factors influencing hospital admission. In addition, we will be able to examine associations with stroke phenotypes as these data are available in the South London Stroke Register dataset.

### Outdoor air pollution and survival following stroke

The association between daily variation in outdoor air pollution levels and stroke is generally clearer for stroke mortality than for hospital admissions. One of the possible explanations is that increases in outdoor air pollution may precipitate death in patients who have already sustained a stroke with resulting compromised respiratory function. Schwartz reported that amongst people dying of stroke on high pollution days, there was a higher level of respiratory disease recorded as a contributory factor compared with people dying of stroke on low air pollution days [26]. Decreased ventilatory function has also been found to be a risk factor for fatal stroke though the mechanism is not clear [27]. Linkage of average outdoor air pollution levels to the South London Stroke dataset will allow examination of the hypothesis that people with stroke living in polluted areas have reduced survival after adjusting for a range of individual level prognostic factors.

## Aim and objectives

The aim of this project is to examine the association between outdoor air pollution levels and stroke using a series of epidemiological studies. The objectives are:

1. To examine acute effects of particulate (PM_10_, black smoke) and gaseous (NO_x_, SO_2_, CO, O_3_) outdoor air pollutants on stroke incidence;

2. To examine if the above effects are modified by age, sex, temperature or socioeconomic deprivation;

3. To examine the differential effects of particulate and gaseous pollutants on incidence using multi-pollutant models and control by matching;

4. To examine the acute effects of outdoor air pollutants on incident stroke phenotypes (pathological, clinical and aetiological subtypes);

5. To examine if stroke incidence rates, and incidence rates by phenotype, are higher in more polluted areas due to the combined effects of acute and chronic exposure;

6. To examine if outdoor air pollution levels influence survival following stroke;

7. To apply Bayesian methodology to the above models to incorporate uncertainty in exposure measurement and obtain more robust effect estimates.

## Design and methods

### Study designs

#### Case-crossover study

Maclure developed the case-crossover design in 1991 as a method for investigating the acute effects of an exposure [28]. This study design offers an elegant alternative to statistical time series modelling for examining short-term environmental effects on health. The case-crossover approach is similar in some conceptual aspects to a matched case-control study. Each individual who has had an event at a particular time is matched to one or more time periods close to the event time when he or she did not have the event (i.e. control periods). The individual's exposures at the time of the event are compared with those of control periods. Each risk set thus comprises a single individual who crosses over between different exposure levels in the case and control time periods. These matched pairs are analysed using conditional logistic regression. A major advantage is that each subject serves as his or her own control and the use of one or more control periods close to the event period means that all covariates that could change slowly over time, e.g. age, socioeconomic status, smoking, are controlled for by matching. Another advantage is that the case-crossover approach allows for control of seasonal variation and time trends in the design phase. It has been demonstrated that even very strong confounding of exposure by seasonal patterns can be controlled for by this method providing control days are adequately sampled to avoid subtle biases [29].

#### Small area level ecological correlation study

We will use this study design to examine if stroke incidence rates, and the incidence rates by phenotype, are higher in more polluted areas due to the combined effects of acute and chronic exposure. 2001 census output areas will be used as the units of analysis. Whilst ecological studies have recognised limitations, the use of small geographical areas as the units for analysis will address some of these limitations. Small area level studies are able to capture fine grain variation in outdoor air pollution levels. In addition, population characteristics, including exposure levels and socioeconomic factors, are likely to be more homogenous within small geographical areas. The analysis should assess spatial autocorrelation in model residuals between neighbouring areas which could bias results and we plan to do this using conditional autoregressive spatial models.[30,31] We have used this study design in several projects examining the impact of environmental and socioeconomic factors on health and healthcare.

#### Cohort study

A cohort study design will be used to examine if outdoor air pollution levels influence survival following stroke. The cohort will comprise patients recorded on the South London Stroke Register. We will use modelled pollution data to attribute an average exposure level to individuals within the cohort. The register tracks residential changes of cohort members within the study area and this information may be used to take account of varying levels of pollution exposure due to changes in residence. Exposure at each location will be weighted by the time spent at that location. Follow up will be from onset of the first stroke to death, end of study period or a move out of the area. The analysis will use standard Cox's proportional hazards modelling to assess the effect of outdoor air pollution on survival.

### Air pollution data

#### Monitored data

Daily outdoor levels of SO_2_, NO_x_, CO, O_3 _and PM_10 _from 1996–2003 will be obtained from Department for Environment Food and Rural Affairs (DEFRA). DEFRA co-ordinates the funding of three automated monitoring networks on behalf of the Government and the devolved administrations – the Automatic Urban Network (AUN), the Automatic Rural Network (ARN) and the Hydrocarbon Network. The number of automatic network sites increased from 74 at the end of 1996 to 116 by June 2003. The increase was mainly in urban sites to provide more comprehensive coverage on air quality in UK cities. The AUN monitors SO_2_, NO_x_, CO, O_3 _and PM_10 _at a variety of urban locations, including urban background locations representative of population exposure for significant periods, and "hotspot" monitoring at urban kerbsides and in the vicinity of industrial sources. The ARN sites mainly monitor O_3 _but also SO_2_, NO_x _and PM_10 _in some cases. Quality control arrangements ensure that measurement precision standards of at least ± 10 μg/m^3 ^for SO_2_, ± 8 μg/m^3 ^for NO_x _and O_3_, ± 0.5 mg/m^3 ^for CO and ± 4 μg/m^3 ^for PM_10 _are achieved at automatic monitoring sites. We will also use data from a non-automatic network, the UK Smoke and Sulphur Dioxide Network. Measurements of daily SO_2 _and black smoke concentrations in urban areas are currently made through this Network, which provides a long-term database for these pollutants to assess trends in concentration and spatial distribution. The use of these data would potentially increase coverage but the limitation is the range of pollutants measured.

#### Modelled data

Validated modelled outdoor air quality data for NO_2 _and PM_10 _will be obtained from the Greater London Authority. Data are available for 1999 and should also become available for 2001. These were produced to inform the Air Quality Strategy for Greater London which deals mainly with ambient, or outdoor, air quality. Air quality was assessed by calculating the amount of air pollution at ground level as an annual average concentration. The model took into account:

- emissions from cars and other vehicles, industrial processes, boilers (such as those used for central heating), construction and various other processes;

- the weather, local geography and topography (shape of the ground's surface, e.g. hills) and the height of the emissions;

- chemical reactions of emissions from these sources in the atmosphere which can create further pollution (i.e. secondary pollution);

- pollution travelling into London from the rest of the UK and overseas (i.e. trans-boundary pollution).

The modelled data were generated at a 10 metre grid square resolution and will be used in the ecological and cohort studies.

### Meteorological data

Data on daily temperature, relative humidity, wind-speed and wind-chill from 1996–2003 will be obtained from the Meteorological Office. These data are required to adjust for potential confounding of the association between air pollution and stroke due to these climate related factors that may vary on a daily basis.

### South London Stroke Register data

The South London Stroke Register is a research register of international standing covering a multi ethnic inner city population of 235,000, which has been ongoing since 1995 [32]. Its aims are to develop chronic disease methodology (case ascertainment, multi level modelling for outcome assessment) and prospectively estimate the impact of disease (incidence and outcome), analyse the predictors of risk and outcome, monitor trends in incidence and survival and estimate and model the resource use and cost of care. The population covers residents in 22 wards of South London. Data are collected prospectively by the registry team, comprising 2 research associates supported by a research stroke physician, a coordinator, trained students and study investigators. Twelve overlapping sources of notification are used to ascertain cases. Hospital surveillance of stroke admissions includes 5 hospitals and checks on health authority activity data outside these hospitals. Death certificates are checked for potential cases that are validated according to clinical registration criteria. ONS notify the register of any patient who dies. Because of the possibilities of under-ascertainment with registers, intense feedback of results and communication with the 140 general practices involved in registering patients is carried out using visits, phone, letter, emails and newsletters. This involves the research associates and a register coordinator. All patients are assessed within 48 hours of the stroke by a study physician. Data collected at the initial assessment are:

Demography – age, sex, ethnicity, socio-economic status, premorbid disability, family history;

Stroke-related risk factors – smoking, alcohol, hypertension, diabetes, atrial fibrillation, transient ischaemic attack, ischaemic heart disease, medications prior to stroke;

Initial impairments – urinary incontinence, motor weakness, loss of consciousness [Glasgow Coma scale], visuospatial neglect, dysphasia and dysphagia;

Stroke phenotypes – pathological (ischaemic, intracerebral haemorrhage, subarachnoid haemorrhage), clinical (Oxfordshire Community Stroke Project classification) [33], aetiological (modified TOAST classification) [34];

Resources – bed type, length of stay, use of investigations, risk factor management.

The Register currently has over 2700 incident cases and is probably the largest European stroke register to date with good phenotype data. All patients are followed up annually.

### Denominator population and socioeconomic deprivation data

Revised 2001 census population counts by sex and five-year age band will be obtained from ONS at the output area level (mean count 297, SD 72) and used for small area level analyses. We will consult local borough councils regarding the accuracy of population counts and residential developments and compare counts with primary care trust population registers derived from the Exeter system before using the data. The 2001 census based Townsend deprivation index and Index of Multiple Deprivation will be used as indices of deprivation at the small area (census output area) level.

### GIS data linkage and modelling

A Geographical Information System (GIS) will be used to link air pollution and meteorological data with health event data [35,36]. GIS is excellent for linkage of data from different spatial frameworks and analytical work will be carried out to examine a number of options for linkage. For linkage of monitored air pollution data, one approach will be to create buffers with varying radii around each monitoring site and assign pollution values to all cases (residential postcodes) located within these buffers. Where buffers overlap, postcodes may be linked to the nearest monitor. Another approach is to either calculate the daily average of all monitoring stations within the South London study area or spatial interpolation within the area to create a pollution surface, from which pollution levels may be assigned to postcodes. A further issue is the selection of monitors for linkage. We will examine the spatial distribution and population coverage of the different types of monitors and consult with DEFRA before deciding on the monitored pollution information to be used. We will use areal interpolation methodology we have refined for interpolating modelled pollution data from grid squares to output areas for small area level analyses [35,36]. For cohort study members, we plan to use buffers of varying radii around residential postcodes to examine the effect of spatially averaged exposure levels, in addition to assigning exposure based on point in polygon methodology. Monitoring stations for meteorological data are generally not co-located with air pollution monitoring stations. We will examine the distribution of these stations and investigate options including buffer-based and spatial interpolation methods as described above before choosing an appropriate method for linking meteorological data to stroke event residential postcodes.

### Statistical modelling and analysis

The case-crossover dataset will be analysed using conditional logistic regression within SAS. Pollutants and meteorological variables will be examined as continuous and as categorical variables. We will examine a range of sampling strategies for obtaining control values and a range of lag days and cumulative lag periods. Effect modification due to sex, age, temperature and socioeconomic deprivation will then be investigated. Differential effects of pollutants will be examined using multi-pollutant models and an alternative method, control by matching, developed by Schwartz [37]. Conditional autoregressive spatial modelling within a Bayesian hierarchical framework will be used for spatial analysis, with expected counts standardised for age and sex, and deprivation included as a covariate. Models will be checked for sensitivity to priors. Cox's proportional hazards modelling will be used for survival analysis, adjusting for a range of prognostic factors. We will then model uncertainty in exposure assessment using a conditional logistic approach within a Bayesian framework based on Bayesian approaches to errors in variables. There are at least four sources of uncertainty in exposure measurement in the acute effects analyses – distance of residential postcode from monitoring site, measurement precision, variation in levels within a day for daily average measures, and variation within a cumulative exposure period.

### Other issues

Ethics approval for research using the South London Stroke Register has been granted by the St. Thomas' Hospital Research Ethics Committee. There will be no patient contact and no intervention involved in this research project.

Air pollution is potentially a modifiable risk factor for stroke. The study will provide robust quantified evidence regarding the effects of outdoor air pollution on stroke incidence, phenotypes and survival. This will provide policy-makers at national and international levels with detailed information and evidence to be used in conjunction with other available evidence when decisions are made regarding national air quality standards and stroke prevention.

The results will be presented at local, national and international meetings and submitted for publication in peer-reviewed journals. Details of the work and results will also be placed on the Public Health GIS Unit's website [38] which regularly attracts interest from an international audience.

### Funding

The project is funded by the Colt Foundation [39]. It commenced in October 2005 and is funded for a two year period.
